# Determination of pharmacokinetic-pharmacodynamic cutoff values of oxytetracycline in calves and adult cattle using population pharmacokinetic modeling

**DOI:** 10.3389/fmicb.2024.1498219

**Published:** 2024-12-04

**Authors:** Esther A. Winter, Ludovic Pelligand, Pierre-Louis Toutain, Peter Lees, Aneliya Milanova, Ronette Gehring

**Affiliations:** ^1^Veterinary Pharmacotherapy and Pharmacy, Department of Population Health Sciences, Utrecht University, Utrecht, Netherlands; ^2^Department of Clinical Science and Services, The Royal Veterinary College, Hatfield, United Kingdom; ^3^Department of Comparative Biomedical Science, The Royal Veterinary College, Hatfield, United Kingdom; ^4^INTHERES, INRAE, ENVT, Université de Toulouse, Toulouse, France; ^5^Department of Pharmacology, Animal Physiology, Biochemistry and Chemistry, Faculty of Veterinary Medicine, Trakia University, Stara Zagora, Bulgaria

**Keywords:** oxytetracycline, cattle, NLME, PK/PD, clinical breakpoint, VetCAST, antimicrobial, BRD

## Abstract

**Introduction:**

A harmonized clinical breakpoint for interpreting antimicrobial susceptibility testing of oxytetracycline in cattle is currently lacking in Europe. This study aimed to establish a pharmacokinetic/pharmacodynamic (PK/PD) cutoff to propose clinical breakpoints, facilitating reliable interpretation of antimicrobial susceptibility results in cattle.

**Methods:**

A meta-analysis of oxytetracycline pharmacokinetic data from 69 cattle was conducted, including 1,730 plasma concentration samples from animals administered 20 mg/kg intramuscularly and/or 20 or 40 mg/kg intravenously. A three-compartment model with two absorption phases was selected, incorporating age as a covariate for clearances and distribution volumes. The PK/PD cutoff was defined as the maximum MIC for which the *f*AUC/MIC index achieves the pharmacodynamic target in 90% of cattle given the standard dosing regimen. The pharmacodynamic index (PDI) target selected was established to 24 h, i.e., the average free plasma concentration of oxytetracycline over the 24-h dosing interval, under steady-state conditions, is equal to the selected MIC.

**Results:**

Simulations indicated a PK/PD cutoff of 2 mg/L in adult cattle and 1 mg/L in calves for intramuscularly administered long-acting products at 20 mg/kg with a 48-hour efficacy duration. The difference is attributed to higher clearance rates in calves.

**Discussion:**

The established PK/PD cutoffs, when used alongside the wild-type bacterial epidemiological cutoff, can aid in setting clinical breakpoints for oxytetracycline, supporting effective antimicrobial therapy in cattle and accounting for age-related pharmacokinetic differences.

## 1 Introduction

Oxytetracycline (OTC) is a broad-spectrum antibiotic, licensed for treatment of infections in food producing animals. In Europe, the European Medicines Agency’s Antimicrobial Advice *ad hoc* Expert Group (AMEG) has ranked tetracyclines as class D antimicrobial drugs. Class D drugs should be used as first line treatments whenever possible (EMA, 2020).

In Europe, OTC is used to treat bovine respiratory disease (BRD) caused by *Mannheimia haemolytica*, *Pasteurella multocida* and *Histophilus somni* (O'Connor et al., 2019; Welling et al., 2020; De Jong et al., 2023). Resistance of these bovine pathogens to OTC has been based on three descriptors of resistance, namely resistance genes, the epidemiological cutoff (ECOFF) and the CLSI (Clinical Laboratory Standards Institute) clinical breakpoint (CBP). EFSA (European Food Safety Authority) has reported resistance to OTC in cattle based on the proportion of resistant isolates, irrespective of the selected cutoff. Their latest report established the resistance to OTC in cattle pathogens in Europe as 3.1, 17.2 and 20.8% for *H. somni*, *M. haemolytica* and *P. multocida,* respectively (Nielsen et al., 2021). VetPath’s resistance monitoring program of the European Animal Health Study Center (CEESA), using the CLSI CBP, reported resistance to tetracyclines of 11.6% for *P. multocida* and 17.6% for *M. haemolytica.* These values were unchanged between 2009 and 2012 and 2015–2016 (De Jong et al., 2023).

Antibiotic drug selection should be based on pathogen susceptibility. The antimicrobial susceptibility test (AST) describes pathogens as susceptible (S) or resistant (R) based on a CBP. This breakpoint is the highest minimum inhibitory concentration (MIC) deemed to be successfully treated at a given dosage regimen of an antibiotic. In the United States, the Veterinary Antimicrobial Susceptibility Testing (VAST) committee, a subcommittee of the CLSI, has determined a CBP for OTC in cattle, for a single intramuscular dose of 20 mg/kg. They set the CBP of OTC at ≤2 mg/L for susceptible and ≥ 8 mg/L for resistant for *M. haemolytica*, *P. multocida* and *H. somni*. CLSI breakpoints may not be applicable in Europe, because of product and dosage differences between Europe and the United States. Currently, in Europe, there is no harmonized CBP for OTC in cattle.

In Europe, the Veterinary Committee on Antimicrobial Susceptibility Testing (VetCAST), a subcommittee of the European Union Committee on Susceptibility Testing (EUCAST), is responsible for establishing the CBP for veterinary pathogens. It is based on three cut-off values: ECOFF, the highest MIC of wild-type bacteria, the pharmacokinetic/pharmacodynamic cutoff (PK/PD_CO_), which is the highest MIC that can be achieved in a given percentage of subjects in a population for a given target value of a PK/PD index (*vide infra*) and a given dose, and the clinical cutoff, which aims to predict sick animals having, or not, the possibility of recovery with the dosage and formulations used. As no appropriate clinical data for the determination of a clinical cutoff are available to determine a clinical cutoff for most veterinary pathogens and antibiotics, the CBP is generally based on the ECOFF and the PK/PD_CO_.

In the VetCAST method, the PK/PD_CO_ is determined by building a PK population (popPK) model and using this in Monte Carlo Simulations, simulating plasma concentration profiles for different dosing regimens. By using a popPK model, the drug exposure variability within the full population is accounted for. A popPK model requirement is the availability of raw data covering the whole population in all its diversity, i.e., different formulations and for animals of differing age, sex, health status etc. corresponding to the different elements of the cattle population likely to receive OTC.

The PK/PD_CO_ is the highest MIC at which a predetermined percentage of the population, usually 90% (Toutain et al., 2017), reaches the predetermined pharmacodynamic target (PDT). This PDT is dependent on the selected target of the pharmacodynamic index (PDI). For tetracyclines (Ambrose et al., 2007), the best PDI is the ratio *f*AUC/MIC, where *f*AUC is the Area Under the Curve of free (i.e., unbound to proteins) plasma concentration for the presumed duration of efficacy of the formulations under investigation. Free plasma AUC concentration is used, as efficacy depends on free drug concentration. In cattle, estimates of OTC protein binding ranged from 31.6 to 71.7% (Pilloud, 1973; Ziv and Sulman, 1972; Nouws et al., 1985a; Lees et al., 2016). The OTC PDT for bacteriostasis of *M. haemolytica* ranged from 19 to 42 h, depending on the growth medium and experimental method (Brentnall et al., 2013; Lees et al., 2018).

Long-acting (LA) OTC products for i.m. administration, containing the hydrochloric salt or dihydrate of OTC, provide sustained plasma concentrations, aimed at achieving average free concentrations equal to the MIC for prolonged periods. This enables long dosing intervals or single dose administration (Mestorino et al., 2016; Brentnall et al., 2013). Recommended dose rates are 20 mg/kg or 30 mg/kg i.m. as a single or repeat dose.

The aim of this study was to determine the PK/PD_CO_ of OTC, administered i.m. in a LA formulation at a dose rate of 20 mg/kg.

## 2 Materials and methods

### 2.1 Data collection

For this meta-analysis, eight data sets were used: three published studies (Mileva et al., 2020; Lees et al., 2018; Clarke et al., 1999), two studies from academic groups (unpublished) and three unpublished studies from pharmaceutical companies. For all data sets times of blood sampling and dose administration were known.

The cumulative data set included 1,730 data points from 69 animals. These included calves (*n* = 28) and adult cattle (*n* = 41), dairy (*n* = 24) and beef (*n* = 45) breeds and male (*n* = 30) and female (*n* = 39) animals. 14 animals had infections, of which 8 were calves in a severe pneumonia model, euthanised after 48 h (Lees, unpublished) and 6 had metritis associated with *Trueperella pyogenes* (Mileva et al., 2020). Eight OTC products were used: one for i.v. administration, the remainder for i.m. administration of LA formulations administered at the licensed dose of 20 mg/kg. In three studies OTC was administered i.m. once; in three two OTC products were administered i.m. with wash-out periods of 7, 10 or 33 days; and in two OTC was first administered i.v. then i.m.

All sampling schedules were rich, with 11 to 27 samples harvested per animal per OTC administration; at least 2 samples were taken within the first hour and at least 7 within 24 h of dosing. Data sets included patient characteristics, such as age (adult or calf), sex, health status, OTC products administered and dairy or beef cattle (summarized in Supplementary Tables S1, S2. All raw data are included in an available Excel file).

#### 2.1.1 Analytical method

All studies used validated methods (High Pressure Liquid Chromatography with ultraviolet detection) to measure plasma OTC concentration, except one study (Clarke et al., 1999), in which a microbiological assay was used. The methods are presented in Supplementary Table S3.

The Lower Limit of Quantification (LLOQ) of the HPLC methods ranged from 0.02 to 0.15 μg/mL and for the microbiological assay it was 0.5 μg/mL. The study with the longest sampling duration after a single administration (up to 168 h after dosing) was the only one with more than one value less than LLOQ (Mileva et al., 2020), with 10 samples less than the limit of 0.15 μg/mL. As concentrations less than LLOQ comprised only 0.7% of total samples (12 of 1,730), these data were discarded; this is the method M1 approach previously described (Beal, 2001). This did not impact on data analysis (Byon et al., 2008).

### 2.2 Data analysis

Data analysis was carried out using Phoenix®WinNonlin®8.3 (Certara, Princeton, New Jersey, USA).

#### 2.2.1 Population modeling

##### 2.2.1.1 Development of the base model

Several structural models (2 or 3 PK compartments with one or two rate constants of absorption from an extravascular site of administration) and several error models were evaluated. Inter-individual variability for the structural parameters was described by an exponential model, conducted using a non-linear mixed effects (NLME) approach. Structural models were compared: (1) using -2LogLikelihood (−2LL) and Bayesian Information Criterion (BIC) with the FOCE-ELS (First-Order Conditional Estimation-Extended Least Squares) engine and; (2) by exploring the diagnostic plots to determine the best-fitting model. With the inclusion of i.v. data, bioavailability (F) could also be determined for extravascular administration; it was estimated using an ilogit transformation, to prevent the bioavailability being estimated greater than 100%. For the error model, a combined additive and proportional model was selected. For the random components of the exponential model, a full Omega matrix (i.e., with both variance and covariance) was selected for parameters associated with absorption and another full Omega matrix was used for disposition parameters (V1, V2, V3, Cl, Cld2, Cld3). The eta and epsilon shrinkages were computed.

##### 2.2.1.2 Covariates

When the base model was established, the impact of possible covariates was explored. Age was first assessed as a possible covariate for clearances (Cl, Cld2, Cld3) and volumes of distribution (V1, V2, V3). Age was a categorical covariate, as not all ages were known, with calves classified as animals less than 6 months old or when the authors declared the animals to be calves. Clearance and volume of distribution are likely to change with age due to changes in blood flow to excretory organs and hepatic enzyme expression. After exploring age as a covariate, formulations and analytical method were added as a combined possible covariate; it was named ‘source’ since the source predictably influences the variability of measured concentration and absorption rate. Several additional covariates and covariate combinations were also explored, based on published studies and mechanistic reasoning, such as health status, breed and sex. These categorical covariates were assessed using an exponential model (Equation 1):


(1) $$\mathrm{stparm}\left(P=tvP*\exp\left(\mathrm{dadul}t_{\mathrm{calfP}}*\left(\mathrm{adul}t_{\mathrm{calf}}==1\right)\right)*\exp\left(nP\right)\right)$$

where P is the PK parameter, tvP is the typical value of the P population, dadult_calfP is the categorical covariate age on P and nP is the eta, i.e., the variability of P in individual animals. Adults and calves are coded in the data sets with ‘0’ for adults (control condition) and ‘1’ for calves.

The impact of adding covariates and covariate combinations was considered by assessing BIC values; Differences in BIC between models of >6 is accepted as “strong” evidence in favor of the model with the lower BIC (Kass and Raftery, 1995). A BIC value of 6.635 was selected for adding a covariate. Diagnostic plots and visual predictive checks were then inspected to confirm or reject inclusion of the covariates. To determine the overall adequacy of the model, Visual Predictive Check (VPC) was made using 500 replicates of each animal and computing a 80% prediction interval.

For the final model, primary PK parameters with covariates were estimated for both i.m. and i.v. data. The secondary PK parameters, Volume of distribution at steady-state (V_ss_), half-life (t_1/2_), AUC, and bioavailability (F), computed from the estimated ilogit and mean residence time (MRT) were also computed. Precision of estimated parameters (CV% and Confidence Intervals) was determined using a bootstrap method with 50 replicates. A precision of 30% for fixed effect and 50% for random effect was accepted.

#### 2.2.2 Model application: PK/PD integration and Monte Carlo simulations

The final model included only the covariate age for volumes of distribution (V1, V2 and V3) and clearances (Cl, Cld2, Cld3) (see Discussion). Monte Carlo Simulations (MCS) were performed with this model for i.m. doses of 10 mg/kg, 20 mg/kg and 30 mg/kg assuming linear pharmacokinetics. The exposure to OTC was computed as the total area under the curve (AUC) from time 0 to 1,000 h post-administration (*n* = 5,000 replicates). By definition, the total AUC after a single dose administration is equal to the AUC over a given dosing interval in steady-state conditions. To account for the unbound drug fraction (*f*), AUCs were converted to *f*AUC by multiplying AUC by the free fraction. Based on previous publications, the percentage plasma protein binding of OTC was set to 50% (Pilloud, 1973; Ziv and Sulman, 1972; Nouws et al., 1985a; Lees et al., 2016).

These AUCs were used to calculate PK/PD indices for doses and dose intervals by dividing the *f*AUC by the dose interval and then by the MICs (0.5, 1, 2, 4 and 8 mg/L). Assuming efficacy and an *in vitro* bacteriostatic action with *f*AUC24h/MIC = 19.1–28 h (Brentnall et al., 2013; Lees et al., 2018), the PK/PD target was fixed at 24 h for a dosing interval of 24 h (or equivalently to 48 h for a dosing interval of 48 h). The quantiles of *f*AUC/MIC distribution were calculated. The PK/PD_CO_ then corresponds to the highest MIC for which 90% of *f*AUC/MIC values are equal to or greater than the Pharmacodynamic Target (PDT). Therefore, the computed PK/PD_CO_ should ensure that the average free plasma concentration during the dose interval, at steady-state, at least equals the selected MIC, whatever the pathogen, for 90% of animals.

## 3 Results

### 3.1 Visual inspection of raw data

All raw data are presented in a Supplementary file.

**Figure 1:**
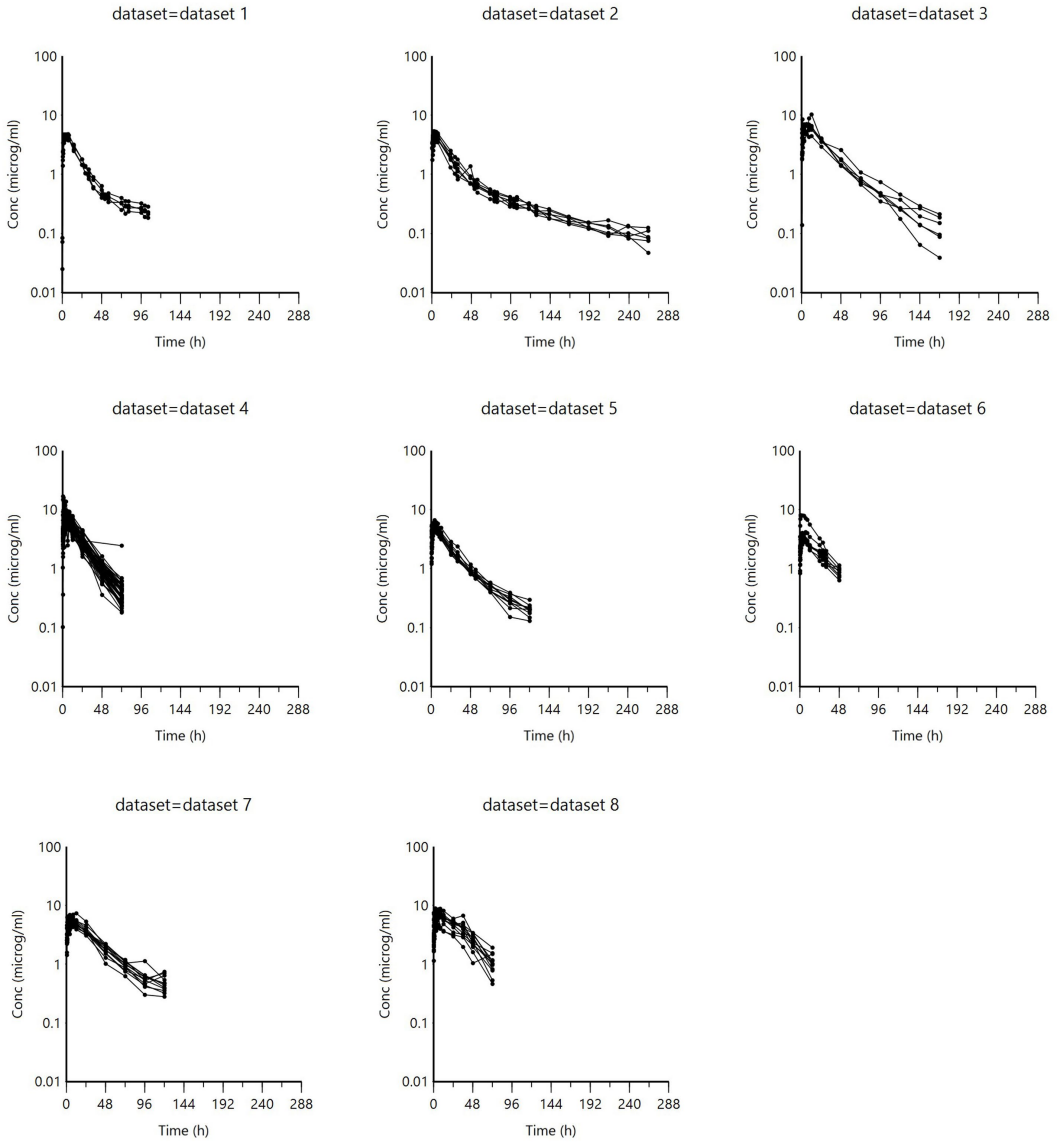
Concentration versus time relationship of all i.m. administrations, grouped by data set. (Data set information is presented in Supplementary Table S1).

**Figure 2:**
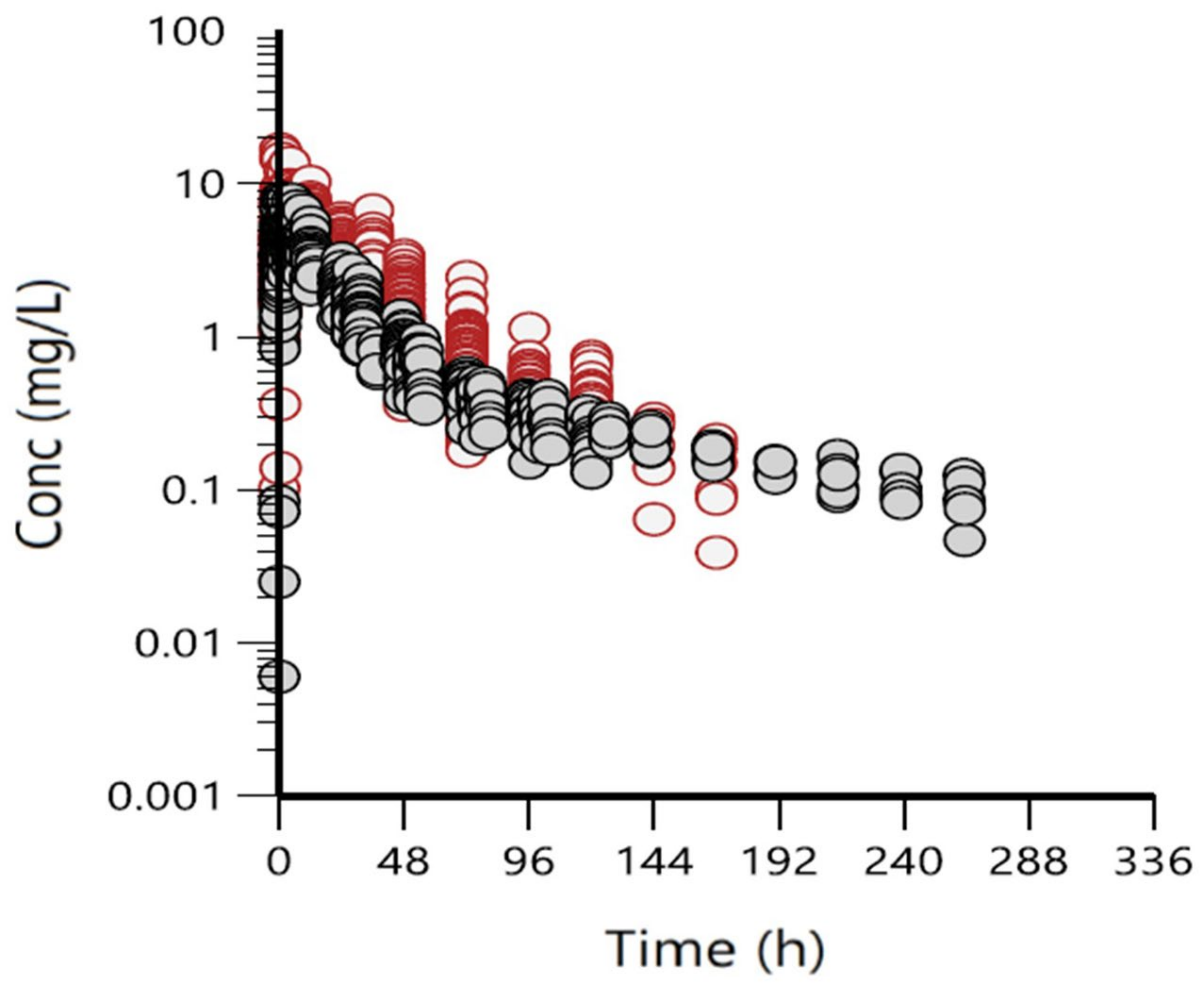
Concentration versus time relationship of all i.m. administrations, grouped by age: red = adult cattle, gray = calves (<0.5 years old).

Spaghetti plots of the i.m. data and data grouped by age are illustrated in Figures 1, 2. The data were also grouped into other possible covariates (health status, race, and sex); these are presented in Supplementary Figure S1. Visual inspection of Figure 2 indicates that calves (age < 6 months) consistently had lower concentrations than adult cattle.

### 3.2 Population modeling

**Figure 3:**
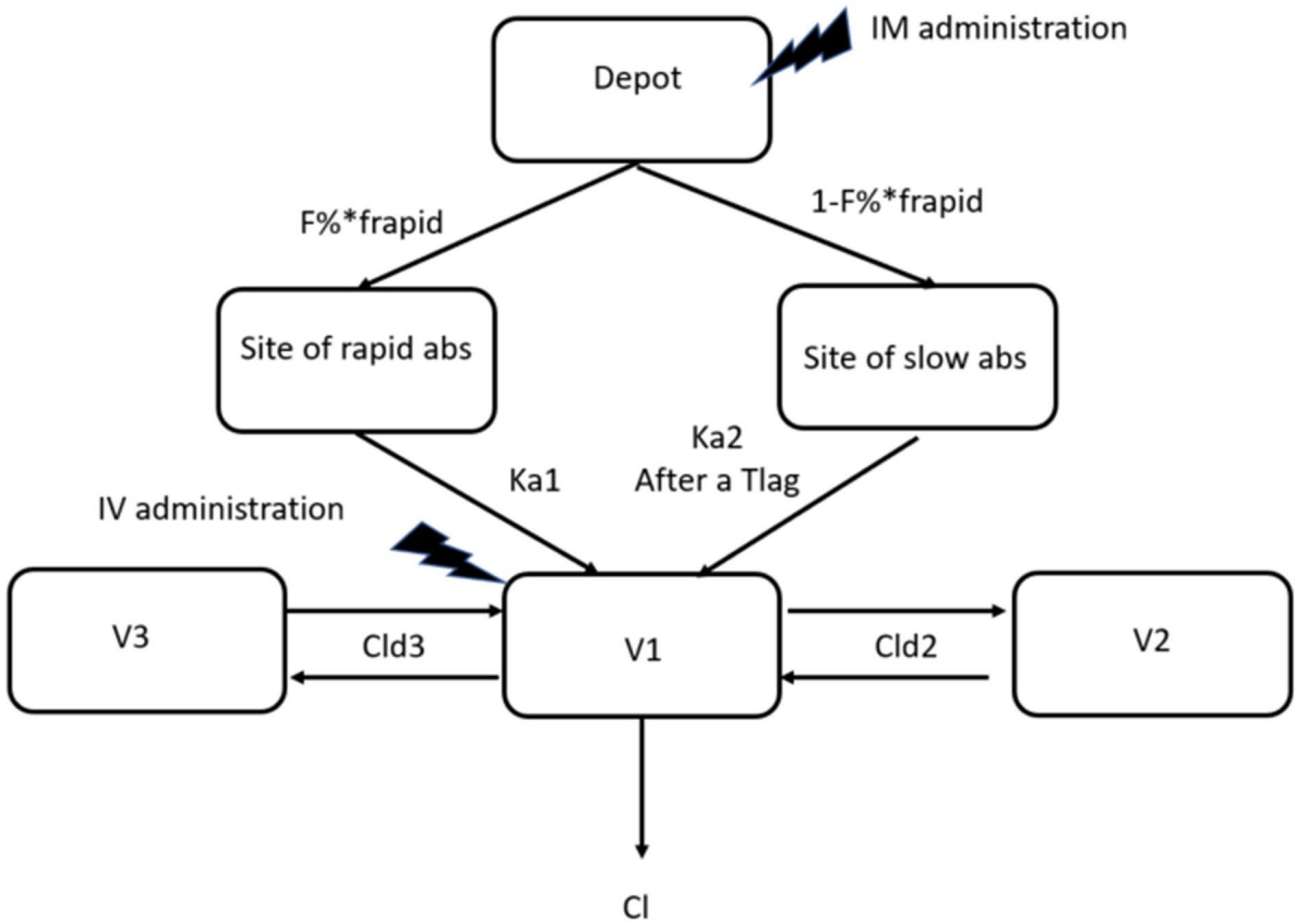
the 3-compartment model selected to model all data sets. F% is the overall bioavailability. F rapid is the fraction of F% that is rapidly absorbed with Ka1, the rapid rate constant of absorption, Ka2 is the slow rate constant and T_lag_ the leg time to absorption between Ka1 and Ka2. Cl is elimination clearance; Cld2 and Cld3 are the rapid and low clearance of distribution to compartment 2 and 3, respectively. V1 is the volume of distribution of the central compartment, V2 and V3 are volumes of distribution of peripheral.

The best fitting structural model was a 3-compartment model with two rate constants of absorption and a combined proportional and additive error model (Figure 3).

**Figure 4:**
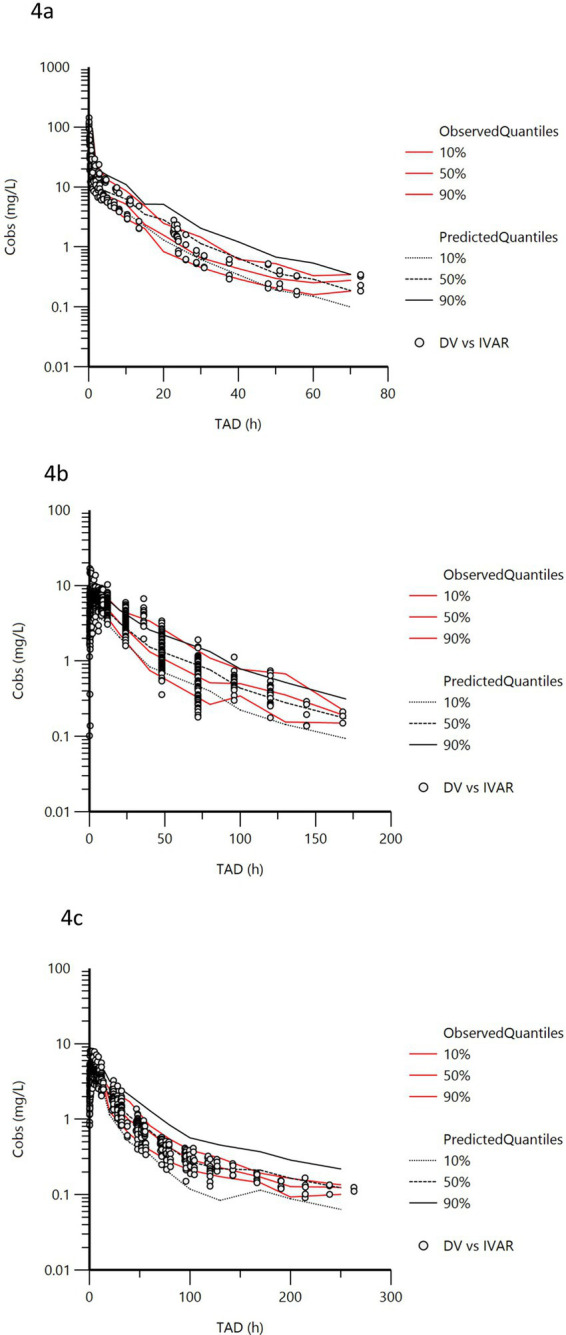
Visual predictive Check (oxytetracycline). VPCs were obtained for 500 replicates. The observed quantiles (10, 50 and 90%) (red lines) are well superimposed on the corresponding predictive check quantiles (black lines) of the observed data. Theoretically, approximately 20% of data (black symbols) should be outside the plotted quantiles. X axis = hours and Y axis = mg/L. (a) intravenous administration, (b) intramuscular administration in adult cows, (c) intramuscular administration in claves (< 0.5 years old).

Adding age as a covariate for clearance (Cl, Cld2, Cld3) and volume of distribution (V1, V2, V3) improved the model fitting, with the lowest BIC of all models evaluated. The Goodness-Of-Fit plots (Supplementary Figure S2) of the final model and the VPC (Figure 4), establish the adequacy of the model.

Typical values of both the single run and bootstrap (*n* = 50), CV% and 2.5 and 97.5% confidence intervals of the parameters evaluated with the bootstrap are presented in Table 1. Estimates of random effects, i.e., Between Subject Variability and eta shrinkage for the full variance/covariance matrix are presented in Table 2.

**Table 1:** Typical values of principal PK parameters and CV% of these estimates determined with bootstrap and stratified by age (*n* = 50).

	Single run		Bootstrap				Definition
					CI (%)		
	Estimates	Units	Median	CV%	2.5	97.5	
tvV1 (adult)	126	mL/kg	132	6.44	116	143	Volume of central compartment (adult)
tvV2 (adult)	914	mL/kg	905	4.12	849	973	Volume of first peripheral compartment (adult)
tvV3 (adult)	2,564	mL/kg	2,235	6.29	2029	2,458	Volume of second peripheral compartment (adult)
tvCl (adult)	62.67	mL/kg/h	65.01	2.67	62.80	68.35	Plasma clearance (adult)
tvCld2 (adult)	485	mL/kg/h	496	5.14	457	549	Clearance of distribution to V2 (adult)
tvCld3 (adult)	19.96	mL/kg/h	21.19	5.31	19.90	23.20	Clearance of distribution to V3 (adult)
tvKa1	0.214	1/h	0.218	4.01	0.207	0.235	first rate constant of absorption corresponding to a Mean Absorption Time of 4.57 h
tvKa2	0.0441	1/h	0.0450	6.36	0.0403	0.0491	second rate of absorption corresponding to a Mean Absorption Time of 22.1 h
tvTlag for ka2	14.96	h	14.62	6.08	13.23	15.74	Lag-time between Ka1 and Ka2
tvFrapid	0.757	ilogit	0.760	2.50	0.725	0.783	Bioavailability of the OTC fraction rapidly absorbed (via ka1) corresponding to a rapid *F* = 68.6%
tvF1	1.281	ilogit	1.314	4.76	1.224	1.422	Total bioavailability (ka1 + ka2) corresponding to a total *F* = 78.6%
dadult_calfCl^a^	0.548	scalar	0.530	5.43	0.478	0.570	Categorical covariate age on Cl
dadult_calfCld2^b^	0.190	scalar	0.186	3.96	0.178	0.201	Categorical covariate on Cld2
dadult_calfCld3^c^	0.293	scalar	0.189	8.20	0.179	0.228	Categorical covariate on Cld3
dadult_calfV1^d^	0.320	scalar	0.314	4.96	0.301	0.353	Categorical covariate on V1
dadult_calfV2^e^	0.159	scalar	0.144	4.69	0.130	0.154	Categorical covariate on V2
dadult_calfV3^f^	0.358	scalar	0.511	6.56	0.436	0.535	Categorical covariate on V3
tvCMultStdev	18.2	%	18.3	6.82	16.0	20.3	Residual: coefficient of variation
stdev0	0.0069	μg/mL	0.0080	4.41	0.0072	0.0083	Residual: additive component

Volumes of distribution and clearances for calves were determined using the covariate “adult_calf”.

a Cl for calves = tvCl * (EXP(dadult_calfCl)) = 108.4 for single run and 110.1 for bootstrap.

b Cld2 for calves = tvCld2 * (EXP(dadult_calfCld2)) = 586 for single run and 598 for bootstrap.

c Cld3 for calves = tvCld3 * (EXP(dadult_calfCld3)) = 26.76 for single run and 25.73 for bootstrap.

d V1 for calves = tvV1 * (EXP(dadult_calfV1)) = 174 for single run and 181 for bootstrap.

e V2 for calves = tvV2 * (EXP(dadult_calfV2)) = 1,071 for single run and 1,045 for bootstrap.

f V3 for calves = tvV3 * (EXP(dadult_calfV3)) = 3,669 for single run and 3,691 for bootstrap.

**Table 2:** Estimates of random effects and shrinkage for the model.

single run	nKa1	nKa2	nF1	nTlag	nFrapid	nV1	nV2	nV3	nCl	nCl2	nCl3
Omega											
nKa1	0.080095										
nKa2	0.103949	0.19011									
nF1	−0.095537	−0.138489	0.331033								
nTlag	−0.02741	−0.02635	0.068231	0.040549							
nFrapid	0.02069	0.038294	−0.05192	−0.01144	0.015948						
nV1	NA	NA	NA	NA	NA	0.527444					
nV2	NA	NA	NA	NA	NA	−0.07716	0.031032				
nV3	NA	NA	NA	NA	NA	−0.07185	0.02222	0.107616			
nCl	NA	NA	NA	NA	NA	0.055107	−0.00525	0.018945	0.039763		
nCl2	NA	NA	NA	NA	NA	0.010349	0.013185	0.041094	0.016754	0.047794	
nCl3	NA	NA	NA	NA	NA	0.080094	0.009562	0.055244	0.045213	0.029476	0.19877
BSV%	28.87	45.76	62.64	20.34	12.68	83.34	17.75	33.71	20.14	22.13	46.89
Correlation											
nKa1	1										
nKa2	0.842394	1									
nF1	−0.58672	−0.55205	1								
nTlag	−0.48093	−0.30017	0.588923	1							
nFrapid	0.578894	0.695451	−0.7145	−0.44997	1						
nV1	NA	NA	NA	NA	NA	1					
nV2	NA	NA	NA	NA	NA	−0.60313	1				
nV3	NA	NA	NA	NA	NA	−0.30158	0.384502	1			
nCl	NA	NA	NA	NA	NA	0.380524	−0.14941	0.289613	1		
nCl2	NA	NA	NA	NA	NA	0.06518	0.34238	0.573001	0.384322	1	
nCl3	NA	NA	NA	NA	NA	0.247366	0.121745	0.377718	0.508565	0.302416	1
Shrinkage	16.7%	20.0%	25.6%	43.9%	18.2%	12.8%	26.0%	44.6%	15.7%	41.1%	21.4%

Distribution and bioavailability parameters are set as a full variance/covariance matrix (absorption rates, lag time and bioavailability) as also are the elimination parameters (volumes of distribution and clearances).

All six fixed effects for covariate age were significantly different from 0, thus supporting their inclusion in the final model (Table 1).

To compare the trait of the LA formulations, the covariate ‘source’ was added to the model for Ka2, the slow absorption rate constant which controls the terminal phase. Typical values are presented in Supplementary Table S4. Ka2 values were similar for all formulations, with one exception (formulation 3, data set 4) for which a slow Ka2 could not be identified. This is likely due to the short sampling period (0 – 72 h), which does not allow full elucidation of the slow absorption phase (see Discussion). Other evaluated covariates, health, sex and breed, were also tested, but did not, in any combination, improve the model.

### 3.3 Monte Carlo simulations and PK/PD cutoff determination

**Figure 5:**
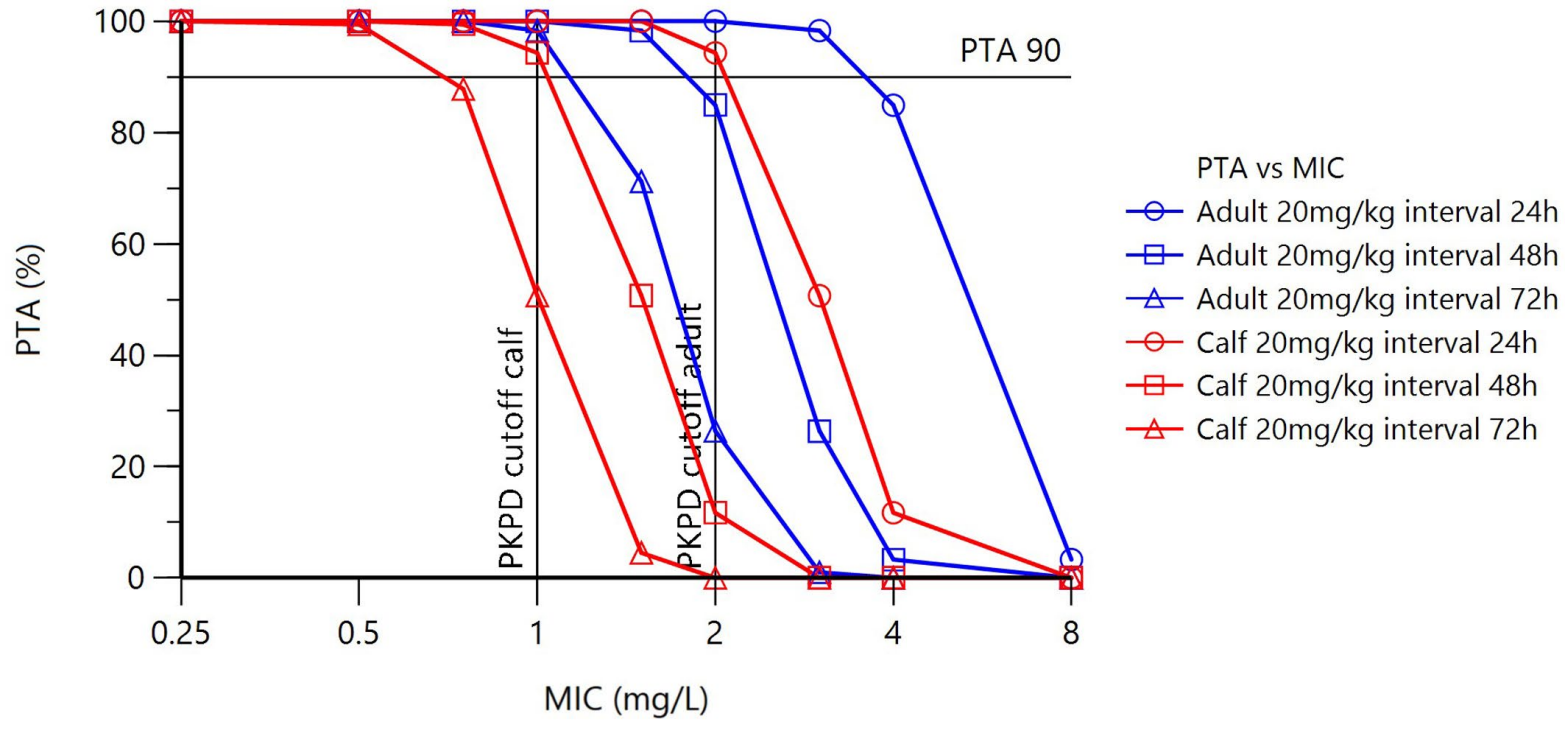
Plot of PTA (%) versus MIC (mg/L). Blue lines = adults, red lines = calves. Open circles = does interval of 24 h, open squares = 48 h and open triangles = 72 h.

Using the final model with covariate age, PTAs for calves and adult cattle for an OTC dosage of 20 mg/kg, with differing dose intervals and different possible MICs, are presented in Table 3 and Figure 5. Several dose intervals were simulated to determine the impact on PK/PD_CO_. Because of the age dependency of plasma clearance, simulations of plasma concentration-time profiles differ for calves and adult cows, resulting in different PTAs. Table 4 presents PK/PD_CO_ values for three dose schedules.

**Table 3:** PTA for different possible MICs for different dosing regimens, with a PDT of 24 h.

MIC (mg/L)	0.0625	0.125	0.25	0.5	1	2	4	8
CALF								
Dose (mg/kg)								
20 q24h	100	100	100	100	100	94.3	11.7	0
20 q48h	100	100	100	100	94.3	11.7	0	0
20 q72h	100	100	100	99.4	50.7	0	0	0
Adult								
20 q24h	100	100	100	100	100	100	84.9	3.3
20 q48h	100	100	100	100	100	84.9	3.3	0
20 q72h	100	100	100	100	98.3	26.4	0	0

**Table 4:** PK/PD_CO_ values for PTA of 90% of a LA product at dosage of 20 mg/kg administered i.m. and dose intervals of 24, 48 and 72 h.

Dose	Dose interval	PD target (*f*AUC/MIC)*	PK/PD_CO_ calf (mg/L)	PK/PD_CO_ adult cattle (mg/L)
20 mg/kg i.m.	24 h	24 h	2.18	3.74
20 mg/kg i.m.	48 h	24 h	1.09	1.87
20 mg/kg i.m.	72 h	24 h	0.73	1.25

*The fAUC used is the fAUC0-1,000h, divided by the dose interval.

The calculated PK/PD_CO_ of 1.09 mg/L in calves at a dose rate of 20 mg/kg and a dose interval of 48 h indicates that, based on the popPK model, 90% of calves in the population will have an average free plasma concentration of 1.09 mg/L over the 48 h of the dosing interval for a dose of 20 mg/kg of OTC administered i.m. in a LA formulation. As MICs are determined by serial 2-fold PK/PD_CO_ dilution, the 1.09 value results in a PK/PD_CO_ of 1 mg/L. The PK/PD_CO_ calculated for adult cattle at a dose of 20 mg/kg and a dose interval of 48 h is 1.87 mg/L, which results in a PK/PD_CO_ of 2 mg/L after rounding up to the next dilution.

## 4 Discussion

In this meta-analysis, two PK/PD_CO_s for OTC, one for calves (1 mg/L) and one for adult cattle (2 mg/L), were calculated using the VetCAST method. They were established for LA OTC products administered at 20 mg/kg, twice with a 48 h interval. For a dose interval of 72 h, the corresponding PK/PD_CO_s were 1 mg/L for adult cattle and 0.5 mg/L for calves. These data are usable to determine CBPs for OTC in cattle with respiratory infections caused by *M. haemolytica, B. bronchoseptica* and *P. multocida.*

Data sets, all rich, from 8 sources were suitable for building a popPK model based on the VetCAST methodology for the determination of PK/PD_CO_. The inclusion of i.v. data sets: (1) enabled identification of the structural 3-compartment model and for a Bayesian estimation of bioavailability (F) of the several formulations administered i.m. and; (2) highlighted the influence of age as a significant covariate on plasma clearance (Cl). Without the i.v. data only a relative clearance (Cl/F) would have been estimated. The age factor would have been confounded with F, because F is related to formulation used and not to a biological factor. Plasma clearance and bioavailability determine AUC, and this variable was used to calculate the OTC PK/PD index (*f*AUC/MIC). Plasma clearance in calves was almost twice that of adult cattle. These age-related differences confirm previous studies and suggest that renal clearance, which is weight dependent, decreases with age (Nouws et al., 1983; Nouws et al., 1985b; Hekman et al., 2022).

For this reason, the covariate ‘age’ was included in the final model calculation of PK/PD_CO_ to adjust for the calf/ adult cattle difference. Had this been discounted, there would be no alternative to declaring, falsely, a lower PK/PD_CO_, impacting negatively on clinical use in adult cattle of this first-line antimicrobial drug. These considerations illustrate the value of NLME-based meta-analysis to calculate PK/PD_CO_.

Data sets were derived from seven LA products, six with an OTC concentration of 20% and one of 30%; they were OTC dihydrate or the hydrate form. In Europe, LA products containing the hydrochloride salt are also licensed. It was assumed that our dataset was sufficiently representative for the cattle population and OTC use in Europe in all its diversity, making it suitable for the establishment of generic PK/PD_CO_ that can be used subsequently to determine a CBP for OTC for cattle for global use. The 3-compartmental model with two absorption rate constants determined with these data confirms previous findings for OTC in cattle (Xia et al., 1983; Nouws et al., 1985b; Meijer et al., 1993), while other studies reported a 2-compartmental model (Kumar and Malik, 2001, 2003; Martín-Jiménez and Riviere, 2002; Mestorino et al., 2007). Between study differences are attributable either to the LLOQ of the analytical method and/or duration of sampling (see Figure 14 for explanation in Toutain and Bousquet-Mélou (2004)). Similar concerns do not arise when data are analyzed with NLME, which analyses uniformly with a 3-compartment model, correctly analyzing unbalanced and censored data. The two absorption phases for OTC have been described previously (Toutain and Raynaud, 1983; Brentnall et al., 2013; Lees et al., 2018).

This study included the covariate age in the final PK/PD model, against the VetCAST recommendation to avoid covariates when determining a single PK/PD_CO_, because age was too large to ignore. This will result in a proposed CBP that differs by one dilution for calves and adult cattle. This situation is managed in an elegant way by EUCAST by the creation of a new class called “I” for” increased exposure”; this class “I “should not be confused with the old class” I” for “intermediary,” which no longer exists in EUCAST terminology. The covariate ‘source’ was not taken into account for the Monte Carlo simulations, so that the large variability in these simulations reflects the diversity of the EU formulations. The relative similarity of the LA formulations investigated is in line with EMA opinion in Annex 7 of the reflection paper on dose review and adjustment of established veterinary antibiotics that differences in absorption, resulting from the composition of LA products, are small because the function of the differing excipients is comparable (CVMP, 2021). The slow absorption of LA formulations is also assumed to result from tissue irritation after i.m. administration (Nouws et al., 1983; Clarke et al., 1999; Romano et al., 2022). In fact, some differences do exist as demonstrated in other studies (Nouws et al., 1985a). However, this could be related rather to the administered dose and subsequent level of irritation than to the formulation (see (Hekman et al., 2022) for explanation).

Covariates explored which did not improve the model were health status, breed and sex, indicating that PK/PD_CO_ was valuable for these factors. In contrast, previous studies did report an effect on OTC PK parameters of infection (Kumar and Malik, 1999) and fever (Kumar and Malik, 2001). This is taken into account in this analysis by computation of a PK/PDco that takes account of an overall variance in which the variability of the health variability is factored.

The PK/PD index that best describes the relation between the PK and PD of OTC and hence best predicts efficacy is *f*AUC/MIC (Ambrose et al., 2007). Also, for all LA products, *f*AUC/MIC is always considered the best PK/PD index (Toutain et al., 2021). To our knowledge, there are only two studies that determined *in vitro* efficacy of OTC for BRD pathogens in calves; *M. haemolytica* (Brentnall et al., 2013; Lees et al., 2018) and *P. multocida* (Lees et al., 2018). For *M. haemolytica* they reported an *f*AUC24h/MIC of 23.2 h from time kill curves in broth and 19.1 h in serum as growth medium (Brentnall et al., 2013; Lees et al., 2018). For *P. multocida* the *f*AUC24h/MIC for a bacteriostatic effect was 28.0 h in serum (Lees et al., 2018). For PK/PD_CO_ estimation 24 h is a standard dosing interval. For LA OTC, the dosing interval is usually longer than 24 h, typically 48 h. In this circumstance, the PDT is simply doubled, i.e., equal to 48 h to ensure that free plasma concentration should equal the MIC for the whole duration of the dose interval to meet the requirement of daily *f*AUC/MIC = 24 h.

CBP should at least equal the ECOFF of the pathogen for treatment of infection by wild type bacteria. However, considering reproducibility of AST results between laboratories, there is a tolerance, under EUCAST, allowing adoption of the ECOFF as the CBP when the PKPD_CO_ is one dilution below the ECOFF. The ECOFF for *M. haemolytica* and *P. multocida* is 2 mg/L (Supplementary Table S5). These can be attained with a dose of 20 mg/kg every 24 or 48 h in adult cattle (Table 4, 1.87 rounded up to the nearest dilution, 2 mg/L) and a dose of 20 mg/kg every 24 h in calves. Assuming dose linearity, the 20 mg/kg dose for 48 h yields the same PK/PD_CO_ as 10 mg/kg with a dose interval of 24 h and 30 mg/kg with a dose interval of 72 h. The present results in adult cattle are in line with the opinion of EMA, who concluded that 20 mg/kg is effective for 24-48 h for susceptible pathogens, but not for less susceptible pathogens such as *M. haemolytica*, for which they recommend redosing after 36-48 h (CVMP, 2021). The EMA did not distinguish between calves and adults in their analysis. The present results indicate that higher doses may be required to reach the ECOFF and treat infections effectively, especially in calves.

The relatively few studies on clinical outcomes suggest that standard doses of OTC in calves might be only marginally more effective than placebo (O'Connor et al., 2016) and less effective than other antibiotics for the treatment of BRD (O'Connor et al., 2019; Jourquin et al., 2022). The only study demonstrating the effectiveness of OTC, administered at a dosage of 20 mg/kg twice with a 48 h interval, was undertaken in calves that were not severely ill, and the applicability of these finding to stressed or clinically ill calves is unknown (Welling et al., 2020). VetCAST cannot consider these clinical data to establish its CBPs because the MIC of the pathogens in these calves is unknown. MICs have consistently been higher in serum than in broth, which may explain the lack of efficacy, as pathogens classified as susceptible in broth could exhibit resistance in a physiological setting (Mead et al., 2019).

The fact that the PK/PD_CO_ calculated for calves in this study (1 mg/L), for an expected duration of action of 48 h, is lower than the ECOFF (Supplementary Table S5) of some pathogens suggests that dosing regimens are inadequate and should be revised. Re-evaluation of the doses, possibly distinguishing calves and adult cattle, and concurrent withdrawal times would be necessary to promote this first choice antibiotic more effectively. A correlate is that companies should also indicate more explicitly the expected duration of action for LA formulations in the product SPC. As suggested previously, it may be necessary to define more than one CBP for OTC in cattle (Toutain et al., 2017).

## 5 Conclusion

The popPK model, constructed from the available datasets, supports a PK/PD_CO_ of 2 mg/L for adult cattle and 1 mg/L for calves, when using a 20% long-acting (LA) formulation at a 20 mg/kg dose rate with a presumed 48 h duration of efficacy. Alternatively, a 10 mg/kg dose every 24 h or a 30 mg/kg dose with a 72 h duration of efficacy may be employed. These dosing regimens are not aligned with all registered OTC products for BRD in Europe. Therefore, product and dosing regimens selection must proceed prudently, especially in calves, to ensure effectiveness.

## Data Availability

The original contributions presented in the study are included in the article/Supplementary material, further inquiries can be directed to the corresponding author.
