# Supplementary material for: Determination of pharmacokinetic-pharmacodynamic cutoff values of oxytetracycline in calves and adult cattle using population pharmacokinetic modeling
Source: Front Microbiol. 2024 Dec 4;15:1498219. doi: 10.3389/fmicb.2024.1498219 (PMC11652485; doi:10.3389/fmicb.2024.1498219)
Supplement: Supplementary file 1 [file Data_Sheet_1.zip › Supplementary files oxytetracycline manuscript.docx]

**Supplementary Material**

**Supplementary Table S1 Characteristics of the cattle used in the eight studies included in the population model of oxytetraycline in cattle. The animals were considered calves if they were < 0.5 years old.**

| **Dataset** | **Data set 1** | **Data set 2** | **Data set 3** | **Data set 4** | **Data set 5** | **Data set 6** | **Data set 7** | **Data set 8** |
| --- | --- | --- | --- | --- | --- | --- | --- | --- |
| **Reference** | Not published, Company 1 | Not published, Company 1 | (Mileva, Karadaev et al. 2020) | Not published, Company 2 | (Lees, Potter et al. 2018) | Not published, Lees | (Achenbach 2000) | (Clarke, Wang et al. 1999) |
| **Products** | Oxyjet 20% LA, Oxyjet 10% PVP | Oxyjet 20% LA, Geomycineject | Tetravet LA | Alamycin LA | Alamycin LA | Alamycin LA | Liquamycin LA, Alamycin LA | BioMycin, OXY Shot LA |
| **Dose** | 20mg/kg i.v.; 6days later 20mg/kg i.m. | 40mg/kg i.v.; 24h later 20mg/kg i.m. | 20mg/kg i.m. | 20mg/kg i.m.; 7 days washout | 20mg/kg i.m. | 20mg/kg i.m. | 20mg/kg i.m. | 20mg/kg i.m. |
| **Time between administrations** | 6 days | 24 hours | na | 7 days | na | na | 33 days | 10 days |
| **population size (N)** | 4 | 6 | 6 | 23 | 10 | 8 | 6 | 6 |
| **age (yr)** | 0.25 | Unknown ^1^ | 3 - 11 | 0.58 - 1.33 | 0.21 – 0.36 | 0.25 | Unknown ^2^ | Unknown ^2^ |
| **Weight (kg)** | 100 - 122 | 70.2 ± 4.5 | 310 - 500 | 270 - 430 | 145 - 204 | unknown | 372±16,8 / 420±17,8 | 295 - 377 |
| **sex** | male | male | female | female | female | male | male | male |
| **breed** | Friesian Holstein | Friesian Holstein | various | various | Aberdeen Angus cross bred | Friesian Holstein | Unknown ^3^ | Polled Hereford |
| **milk / meat breed** | milk | milk | milk | meat | meat | milk | meat | meat |
| **health status** | healthy | healthy | Sick (metritis) | healthy | healthy | Sick (pneumonia) | healthy | healthy |

Footnotes:

1: the age of the cattle in this data set is unknown, but it is mentioned to be calves. It is known to be non-ruminant animals. This fact plus the weight confirms that these animals are calves.

2: the age of the cattle in these data sets are unknown, but as they mentioned to be steers, it is assumed to be adult cattle. The weights also suggest these are adults.

3: the breed of the cattle in this study is unknown, but it is mentioned that it is meat cattle.

**Supplementary Table S2 Registration data of the products used in the studies included in the meta-analysis**

| **Product** | **Salt form of oxytetracycline** | **Concentration** | **Pharmaceutical Company** | **Registered route of administration** |
| --- | --- | --- | --- | --- |
| Oxyject 20 LA | Dihydrate | 200 mg/ml | Dopharma | i.m. |
| Oxyject 10 PVP | Hydrochloride | 100 mg/ml | Dopharma | i.m. |
| Geomycineject | Hydrochloride | 100 mg/ml | Dopharma | i.m. |
| Tetravet LA | Dihydrate | 200 mg/ml | Ceva Sante | i.m. |
| Alamycin LA | Dihydrate | 200 mg/ml | Norbrook | i.m. |
| Biomycin | Amphoteric / anhydrous | 200 mg/ml | Boehringer Ingelheim | i.m., s.c., i.v. |
| OxyShot LA | ? | 300 mg/ml | Generic? | i.m. |
| Liquamycin LA | Amphoteric / anhydrous | 200mg/ml | Zoetis | i.m., s.c., i.v. |

**Supplementary Table S3** **Analytical characteristics per dataset included in the meta-analysis.**

LLOQ = lowest limit of quantification.
BQL = below quantification limit.

| **Dataset** | **Data set 1** | **Data set 2** | **Data set 3** | **Data set 4** | **Data set 5** | **Data set 6** | **Data set 7** | **Data set 8** |
| --- | --- | --- | --- | --- | --- | --- | --- | --- |
| **Analytical method** | HPLC | HPLC | HPLC | HPLC | HPLC | HPLC | HPLC | microbiological assay |
| **LOQ (microg/ml)** | 0.02 | 0.02 | 0.15 | 0.1 | 0.1 | 0.1 | 0.1 | 0.5 |
| **Values below LLOQ (N)** | 1 | 0 | 10 | 1 | 0 | 0 | 0 | 1 < LLOQ and 1 missing |
| **Plasma samples (N)** | 200 | 228 | 96 | 598 | 180 | 128 | 132 | 168 |
| **Sampling time after dose (h)** | 0 - 72 | 0 - 288 | 0 - 168 | 0 - 72 | 0 - 120 | 0 - 48 | 0 - 120 | 0 - 72 |

**Supplementary Table S4 Typical values of the primary PK parameters of the model with ‘source’ added as covariate for Ka2.**

Source code 0 : Data set 3
Source code 1 : Data set 1
Source code 2 : Data set 2
Source code 3 : Data set 4
Source code 4 : Data set 5 and 6
Source code 5 : Data set 8
Source code 6 : Data set 7

The tvKa2 are calculated with dSource results.

| Parameter | Estimate | Units |
| --- | --- | --- |
| dadult_calveCL | 0.534111 |  |
| dadult_calveCl2 | 0.184346 |  |
| dadult_calveCl3 | 0.175701 |  |
| dadult_calveV1 | 0.309045 |  |
| dadult_calveV2 | 0.143557 |  |
| dadult_calveV3 | 0.517598 |  |
| dSource1 | -0.18684 |  |
| dSource2 | -0.15919 |  |
| dSource3 | 3.388719 |  |
| dSource4 | -0.36763 |  |
| dSource5 | 0.673124 |  |
| dSource6 | -0.24806 |  |
| tvV1 | 132.8388 | ml/kg |
| tvV2 | 1004.522 | ml/kg |
| tvV3 | 2075.362 | ml/kg |
| tvCl | 62.74012 | ml/(kg*h) |
| tvCl2 | 502.7361 | ml/(kg*h) |
| tvCl3 | 21.03993 | ml/(kg*h) |
| tvKa1 | 0.219401 | 1/h |
| tvKa2 Data set 3 | 0.045297 |  |
| tvKa2 Data set 1 | 0.037577 |  |
| tvKa2 Data set 2 | 0.038630 |  |
| tvKa2 Data set 4 | 1.342053 |  |
| tvKa2 Data set 5 and 6 | 0.031362 |  |
| tvKa2 Data set 8 | 0.088797 |  |
| tvKa2 Dataset 7 | 0.035345 |  |
| tvTlag | 15.56646 | h |
| tvF1 | 1.300078 |  |
| tvFrapid | 0.782794 |  |
| tvCMultStdev | 0.1833 |  |
| stdev0 | 0.008017 |  |

**Supplementary Table S5 ECOFF values (mg/L) of the organisms against oxytetracycline.**

The blue parts represent the wild type. Data will be published on EUCAST.

| **Organism** | **.002** | **.004** | **.008** | **.016** | **.03** | **.06** | **.12** | **.25** | **.5** | **1** | **2** | **4** | **8** | **16** | **32** | **64** | **128** | **256** | **>512** | **ECOFF** |
| --- | --- | --- | --- | --- | --- | --- | --- | --- | --- | --- | --- | --- | --- | --- | --- | --- | --- | --- | --- | --- |
| *Bordetella bronchiseptica* | 0 | 0 | 0 | 0 | 0 | 0 | 48 | 247 | 155 | 20 | 26 | 3 | 2 | 0 | 0 | 13 | 0 | 0 | 0 | 1 |
| *Mannheimia haemolytica* | 0 | 0 | 0 | 0 | 0 | 0 | 0 | 16 | 15 | 32 | 2 | 6 | 0 | 0 | 2 | 6 | 12 | 0 | 0 | 2 |
| *Pasteurella  multocida* | 0 | 0 | 0 | 0 | 0 | 0 | 0 | 17 | 31 | 28 | 13 | 2 | 1 | 1 | 5 | 3 | 3 | 0 | 0 | 2 |

**Supplementary Figure S1 Raw data plots of i.m. administration of 20 mg/kg.**

Different datasets are marked by color, to indicate possible covariates.

Graph A (health status): red = healthy animals; grey = sick animals

Graph B (sex): red = female; grey = male

Graph C (breed): red = beef; grey = dairy

**Supplementary Figure S2**

**Goodness-of-fit plots supporting the 3-compartment structural model with a combined proportional and additive error model and two absorption rates.**

Figure S2A: DV vs IPRED. Arithmetic and logaritmic plot of the dependent variable (DV) (i.e. plasma concentration) versus the individual predicted plasma concentration (IPRED).

Figure S2B: DV vs PRED. Arithmetic and logaritmic plot of the dependent variable (DV) (i.e. plasma concentration) versus the predicted plasma concentration for the population (PRED).

Figure S2C: CWRES vs Time After Dose (TAD) and Time. Plot of Conditional Weighted Residuals (CWRES) versus time after dose and time.

Figure S2D: CWRES vs Qqplot.

**Supplementary Data S1**

**Phoenix code for the final model**

# Green sentences are comments and all start with #

test(){

#3-cp model with 2 sites of absorption with Ka1 and Ka2

deriv(A1 = - (Cl * C)- (Cl2 * (C - C2))- (Cl3 * (C - C3)) + (Abs1 * Ka1) + (Abs2 * Ka2))

# Amount (A0) collected here allow to assess the overall bioavailability (urine and other modalitiees of elimination). By integrating this ODE, you can compute, for a given time, the total ampount of OTC that has been eliminated

urinecpt(A0 = (Cl * C))

#peripheral amount

deriv(A2 = (Cl2 * (C - C2)))

deriv(A3 = (Cl3 * (C - C3)))

#sites of absorption

deriv(Abs1 = - (Abs1 * Ka1))

deriv(Abs2 = - (Abs2 * Ka2))

#give your IPRED that will be used for MCS and VPC (IPRED take into account THETAS & OMEGA but not SIGMA)

C = A1 / V1

#this is to compute individual AUC (total or partial)

#this is to compute the total AUC with IPRED (no noise) in a supplementary table with a time long enough to be at the infinity

deriv(AUCtot=C)

#this is a series of partial AUC to show how to code them; To compute partial AUC from t=0 to t=12h, from t=12 to t=24deriv(AUCt1_t2 or AUC 0 to 24h; edit the following code for any other partial AUC: (t>t1)*(t<t2)*C). Integrating this ODE, you can measure partial AUC at different time

deriv(AUCt12 = (t>0)*(t<12)*C)

deriv(AUCt12_24 = (t>12)*(t<24)*C)

deriv(AUCt24 = (t>0)*(t<24)*C)

# covariate (Categorical) route of administration coded 0 for IV and 1 for IM (to add a column in data sheet). I add here this covariate to stratify my VPC by route of aministration. Other covariates are Age (cow vs calf), source (i.e trials, 0=Bulgaria, 1 Dopharma1, 2=Dopharma2; 3=Norbrook, 4=RVC; 5=Clarke and 6=Achenbach) and health status (0=healthy, 1=disease)

covariate(Route_of_admin())

covariate(Source())

covariate(adult_calve())

covariate(code_health())

#plasma clearance in cow is lower than in calves (see riviere & papich) and I add a covariate Type coded as 0=cow and 1= calf meaning that THETA typical for clearance is for cow and to compute plasma clearance in calf, you have to multiply by exp(type1)

# Rem: when a covariate is caterogical, there is double bracket otherwise a single bracket

dosepoint(A1, idosevar = A1Dose, infdosevar = A1InfDose, infratevar = A1InfRate)

C2 = A2 / V2

C3 = A3 / V3

error(CEps = 0.00801702289086897)

#CObs computed with the add+multiplicative error term parametrised as mix ratio (see documentation) allowing to model CMultStdev that is a fixed effect (you can even change its name)

observe(CObs = C + CEps * sqrt(1 + (C)^2 * (CMultStdev/sigma())^2))

#There is two sites of absorption with an initial partition of the bioavailable dose between these two sites. Frapid is the site from which absorption is rapid (Ka1) and 1-Frapid the site from which the absorption is slowr (Ka2); In addition, there is a delay to absorption to site 2 described by Tlag . Bioavailability is include in the model as F1)

dosepoint(Abs1, bioavail = (Frapid*F1), idosevar = Abs1Dose, infdosevar = Abs1InfDose, infratevar = Abs1InfRate)

#i include here the bioavailability as F1 (the same for the two fraction because an F2 for the slow fraction did not improve the fitting)

dosepoint(Abs2, tlag = (Tlag), bioavail = ((1-Frapid)*F1), idosevar = Abs2Dose, infdosevar = Abs2InfDose, infratevar = Abs2InfRate)

# covariate for the plasma clearance because AUC/MIC is the PK/PD index and AUC is only controled by plasma clearance and a major effect of age of health status could lead to promote different CBP. I also add the covariate age for CLD2, CLD3 and the different volumes because this improved the VPC (but only IV data from calf in this data set)

stparm(Cl = tvCl * exp(dadult_calveCL*(adult_calve==1)) * exp(nCl))

stparm(Cl2 = tvCl2*exp(dadult_calveCl2*(adult_calve==1)) * exp(nCl2))

stparm(Cl3 = tvCl3 *exp(dadult_calveCl3*(adult_calve==1))* exp(nCl3))

stparm(V1 = tvV1 * exp(dadult_calveV1*(adult_calve==1))* exp(nV1))

stparm(V2 = tvV2 * exp(dadult_calveV2*(adult_calve==1))*exp(nV2))

stparm(V3 = tvV3 * exp(dadult_calveV3*(adult_calve==1))* exp(nV3))

#Ka1 is the rapid rate constant of absorption and Ka2 the slow one. I add a covariate for Ka2 because, a priori, all formulations were not equivalent especially the Norbrook formulation for which the terminal slope decreased more rapidly than for other formulations

stparm(Ka1 = tvKa1 * exp(nKa1))

stparm(Ka2 = tvKa2 * exp(nKa2))

#stparm(Ka2 = tvKa2* exp(dSource1*(Source==1)+dSource2*(Source==2)+dSource3*(Source==3)+dSource4*(Source==4)+dSource5*(Source==5)+dSource6*(Source==6 ))* exp(nKa2))

#stparm(Ka2 = tvKa2* exp(dadult_calveKa2*(adult_calve==1))* exp(nKa2))

#Tlag for Ka2

stparm(Tlag = tvTlag * exp(nTlag))

#this is the ilogit transformation for the bioavailability parameter preventing F>1, what is computed is the ilogit of F and f is back computed as a secondary parameters

stparm(F1 = ilogit(tvF1+nF1))

stparm(Frapid = tvFrapid * exp(nFrapid))

# this is the multiplicative component of the error term. it is a fixed effect and hence, editable . I explored the influence of the covariate “source” and I also added a random component reflecting the BSV of coefficient of variation but this does not improve the model.

stparm(CMultStdev = tvCMultStdev)

#fixed effect for the covariate adult vs calve for plasma clearance and healthy vs disease

fixef(dadult_calveCL(enable=c(0)) = c(,0.534111088449228, ))

fixef(dadult_calveCl2(enable=c(0)) = c(,0.184346396087424, ))

fixef(dadult_calveCl3(enable=c(0)) = c(,0.175701331817129, ))

#fixef(dadult_calveKa2(enable=c(0)) = c(,0.1, ))

fixef(dadult_calveV1(enable=c(0)) = c(,0.309044858247043, ))

fixef(dadult_calveV2(enable=c(0)) = c(,0.143556943658068, ))

fixef(dadult_calveV3(enable=c(0)) = c(,0.517598035063096, ))

#fixef(dcode_health(enable=c(0))=c(,-0.0205427544245676,))

#fixed effect for the covariate "source" on ka2

#fixef(dSource1(enable=c(0)) = c(,-0.186838812209557, ))

#fixef(dSource2(enable=c(0)) = c(,-0.159189860014275, ))

#fixef(dSource3(enable=c(0)) = c(,3.38871946679837, ))

#fixef(dSource4(enable=c(0)) = c(,-0.367634074806818, ))

#fixef(dSource5(enable=c(0)) = c(,0.673124425463354, ))

#fixef(dSource6(enable=c(0)) = c(,-0.248056670107283, ))

#initial values for fixed effects

fixef(tvV1 = c(, 132.838831474651, ))

fixef(tvV2 = c(, 1004.52236535283, ))

fixef(tvV3 = c(, 2075.36183012489, ))

fixef(tvCl = c(, 62.7401186952323, ))

fixef(tvCl2 = c(, 502.736104036558, ))

fixef(tvCl3 = c(, 21.0399345131402, ))

fixef(tvKa1 = c(, 0.219400802303474, ))

fixef(tvKa2 = c(, 0.0452968209733835, ))

fixef(tvTlag = c(, 15.5664560034106, ))

fixef(tvF1 = c(, 1.30007777552074, ))

fixef(tvFrapid = c(, 0.782793906377171, ))

fixef(tvCMultStdev = c(, 0.183300081841436, ))

#full matrix for parameters associated to absorption and bioavailability

ranef(block(nKa1,nKa2,nF1,nTlag,nFrapid)=c(0.079597901,

0.088514211,0.13622132,

-0.082454774,-0.10164019,0.31693713,

-0.029366528,-0.030396783,0.056071371,0.037098993,

0.019723966,0.028331684,-0.036415566,-0.01164975,0.014524523))

#this is the full matrix OMEGA for paramaters of disposition

ranef(block(nV1, nV2, nV3, nCl, nCl2, nCl3) = c(0.57482705,

-0.05501473,0.029307278,

-0.061500255,0.023373703,0.083632638,

0.075617724,-0.0050969624,0.010222822,0.043768506,

0.026133022,0.011590842,0.036153015,0.014640673,0.049606297,

0.12279538,0.00095281908,0.036593083,0.058591329,0.023637234,0.25680111 ))

#this is to compute bioavalability & other secondary parameters as as typical values

#bioavailability from F1

secondary(F1=exp(tvF1)/(1+exp(tvF1)))

#secondary parameters for the IV (no covariate hence for adult cow)

secondary(tvKe=tvCl/tvV1)

secondary(tvK12=tvCl2/tvV1)

secondary(tvK13=tvCl3/tvV1)

secondary(tvK21=tvCl2/tvV2)

secondary(tvK31=tvCl3/tvV3)

secondary(tvVss=tvV1+tvV2+tvV3)

secondary(tvMRTIV=tvVss/tvCl)

#block to compute macroparameters for a 3-cpt model

secondary(a0=tvKe*tvK21*tvK31)

secondary(a1=tvKe*tvK31+tvK21*tvK31+tvK21*tvK13+tvKe*tvK21+tvK31*tvK12)

secondary(a2=tvKe+tvK12+tvK13+tvK21+tvK31)

secondary(p=a1-a2^2/3)

secondary(q=2*a2^3/27-a1*a2/3+a0)

secondary(r1=(-(p^3/27))^0.5)

secondary(r2=2*r1^0.3333)

secondary(PHI=acos(-q/(2*r1))/3)

secondary(root1=-(cos(PHI)*r2-(a2/3)))

secondary(root2=-(cos(PHI+2*3.14159/3)*r2-a2/3))

secondary(root3=-(cos(PHI+4*3.14159/3)*r2-a2/3))

root1

root2

root3

#to compute Alpha>Beta>gamma otherwise A, B and C are false

secondary(tvAlpha=root1>root2 &&root1>root3 && root2>root3?root1:root2)

secondary(tvBeta=root1>root2 &&root1>root3 && root2>root3?root2:root3)

secondary(tvGamma=root1>root2 &&root1>root3 && root2>root3?root3:root1)

secondary(tvHL_alpha=ln(2)/tvAlpha)

secondary(tvHL_Beta=ln(2)/tvBeta)

secondary(tvHL_Gamma=ln(2)/tvGamma)

# to compute HL for the two Ka (flip flop for Ka2 regarding the classical beta phase but not th very late terminal phase that was detected here

secondary(tvHL_Ka1=ln(2)/tvKa1)

secondary(tvHL_Ka2=ln(2)/tvKa2)

#to edit to name alpha>beta>gamma to compute consistently A, B and G

#for a dose of 20mg/Kg : to edit for another dose

Dose=20000

secondary(tvA=((Dose)/tvV1)*((tvK21-tvAlpha)/(tvAlpha-tvBeta))*(tvK31-tvAlpha)/(tvAlpha-tvGamma))

secondary(tvB=((Dose)/tvV1)*((tvK21-tvBeta)/(tvBeta-tvAlpha))*(tvK31-tvBeta)/(tvBeta-tvGamma))

secondary(tvC=((Dose)/tvV1)*((tvK21-tvGamma)/(tvGamma-tvBeta))*(tvK31-tvGamma)/(tvGamma-tvAlpha))

}
